# Supplementary figures and images for: Drosophila CG17003/leaky (lky) is required for microtubule acetylation in early germ cells in Drosophila ovary
Source: PLoS One. 2022 Nov 7;17(11):e0276704. doi: 10.1371/journal.pone.0276704 (PMC9639842; doi:10.1371/journal.pone.0276704)

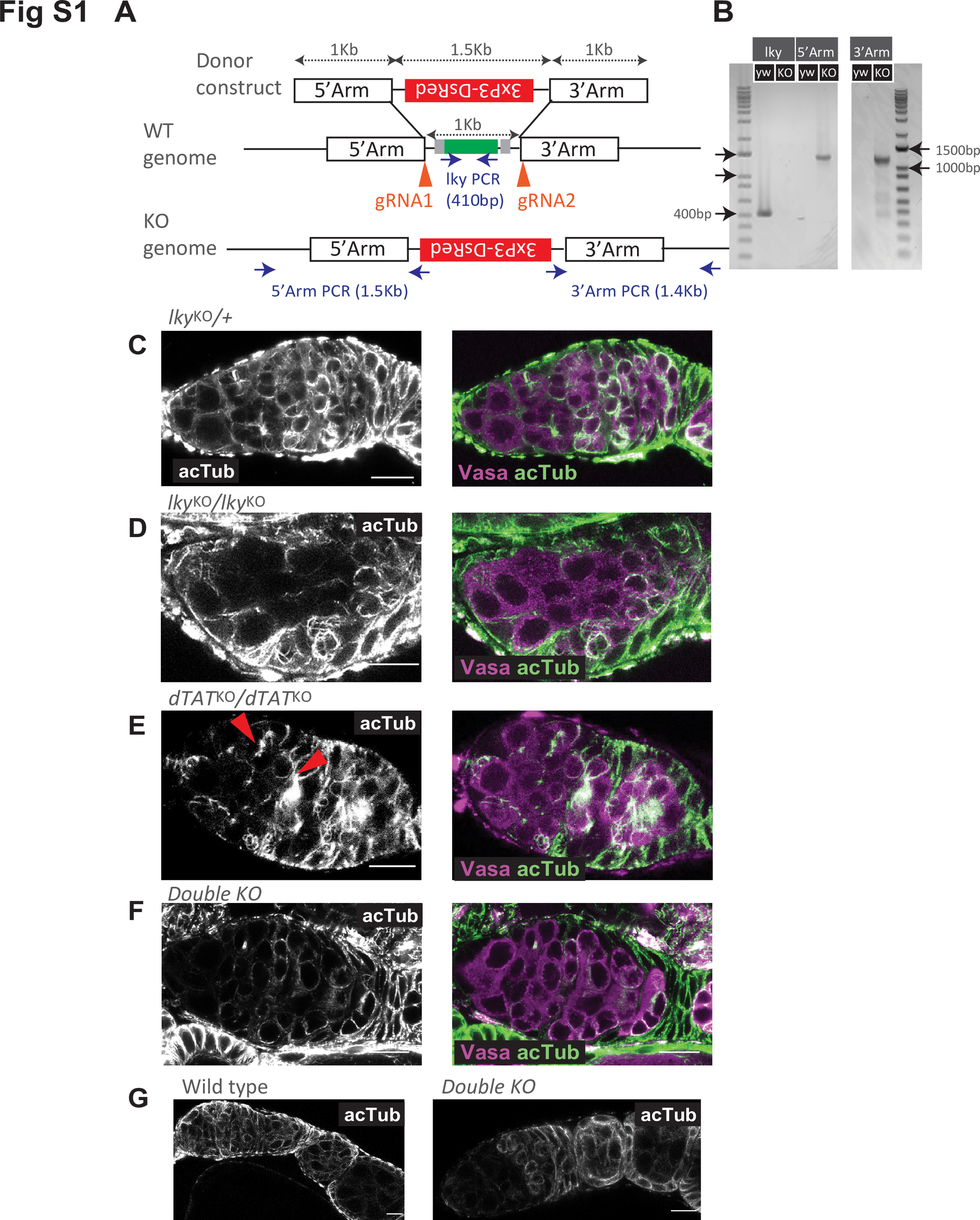

Supplement: S1 Fig — A) A schematic of the construct used to generate lkyKO flies. B) Validation of the KO genotype by PCR. C-F) IF staining for acTub and Vasa of germaria of the indicated genotypes. “double KO” = lkyKO/lkyKO;; dTATKO/dTATKO. G) A representative image of a tip of an ovariole of wild type control (yw) and Double KO showing remaining acTub in later stages of germline and FCs. Flies were used at 0–7 days post-eclosion. Scale Bars; 10μm. Flies were used at 0–7 days post-eclosion. Scale Bars; 10μm. (TIF) [file pone.0276704.s001.tif]

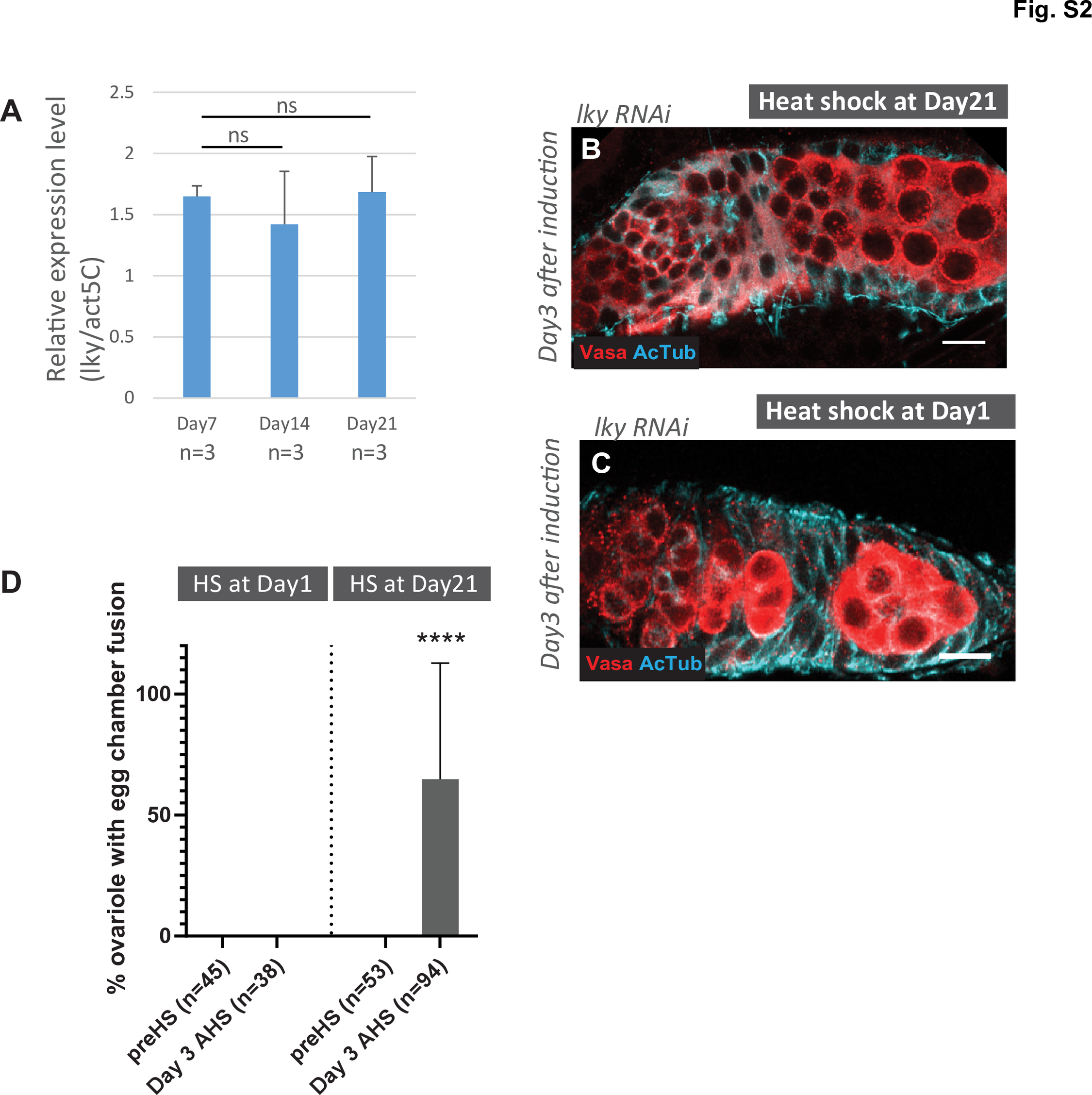

Supplement: S2 Fig — A) A graph shows level of lky mRNA relative to act5C mRNA (lky/act5C) in germaria isolated from indicated ages. Wildtype (yw) flies were used (See Methods for details). B-C) Representative images of germaria of IF staining for acTub (cyan) and Vasa (red). lky RNAi was induced by heat shock (HS) before (1 day post-eclosion) or after aging animals (21 days post-eclosion). D) A graph shows frequency of ovarioles with egg chamber fusion after heat shock at the indicated ages. Adjusted P values from Šidák’s multiple comparisons test are shown. For all data points, a minimum of 20 ovaries were used. Scale bars; 10μm. (TIF) [file pone.0276704.s002.tif]

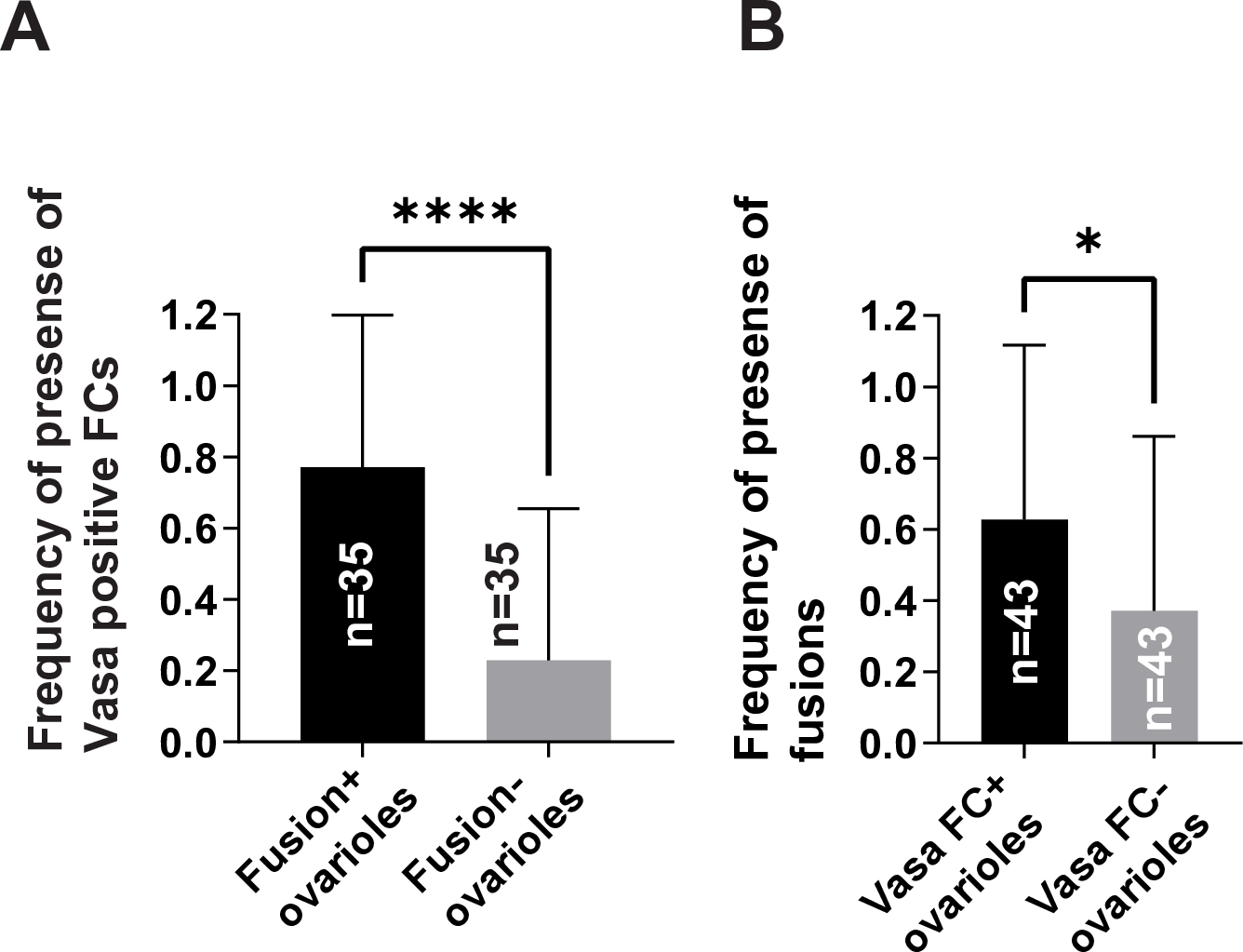

Supplement: S3 Fig — A) Graph shows comparison of frequency of ovarioles with Vasa positive FCs within fusion positive vs. fusion negative ovarioles. B) Graph shows comparison of frequency of ovarioles with fusion within ovarioles with or without Vasa positive FCs. Flies were used at day14 post-eclosion. P values from Student’s t-test are shown. For all data points, a minimum of 20 ovaries were used. (TIF) [file pone.0276704.s003.tif]

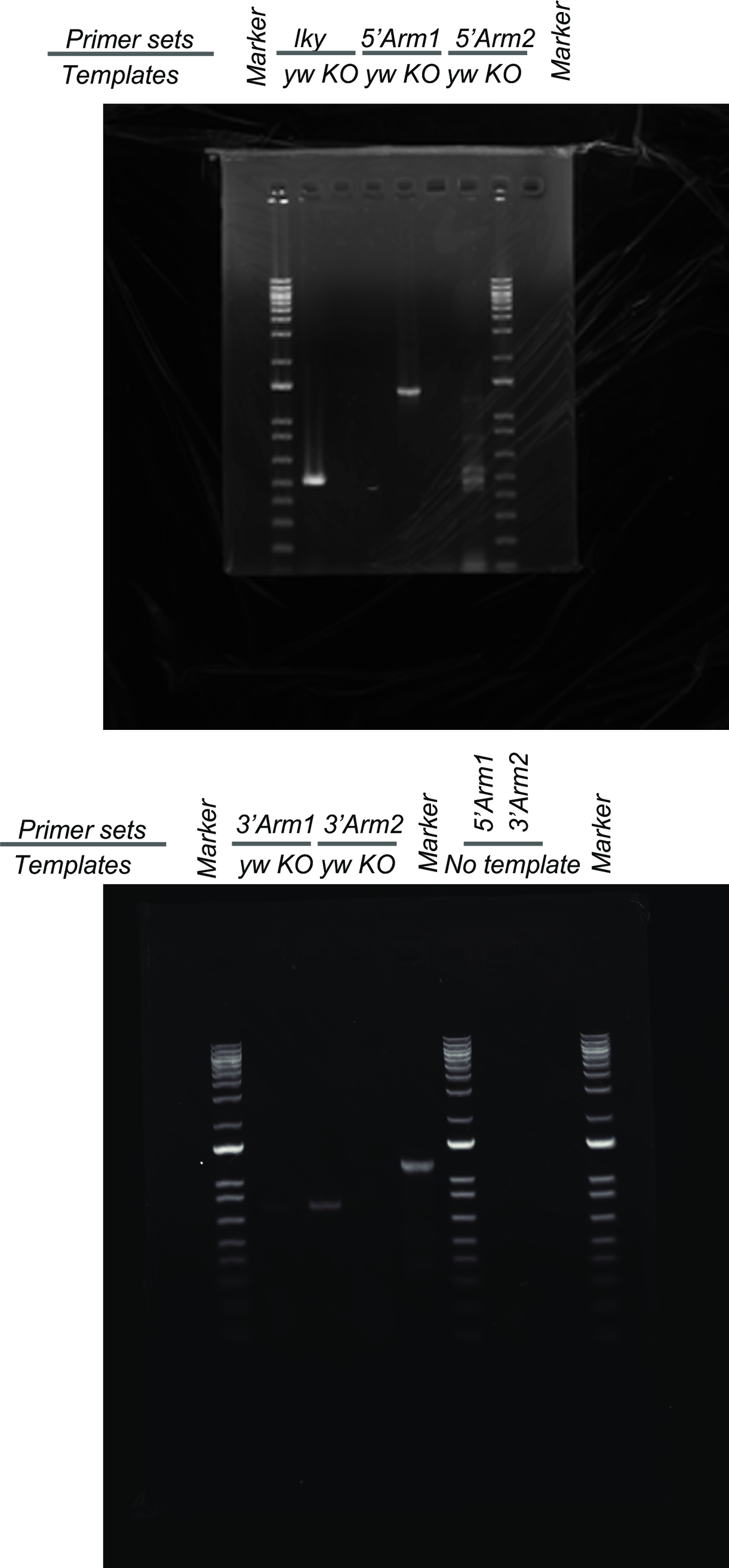

Supplement: S1 Raw images — (TIF) [file pone.0276704.s005.tif]
